# First Description of *Mycoplasma agalactiae* Anatomical Localization in Naturally Infected Hard Ticks (*Rhipicephalus bursa*)

**DOI:** 10.3390/microorganisms12071390

**Published:** 2024-07-09

**Authors:** Sergio Migliore, Lucia Condorelli, Paola Galluzzo, Lucia Galuppo, Angelica Corrente, Elvio Lepri, Anne Ridley, Guido Ruggero Loria, Roberto Puleio

**Affiliations:** 1 Istituto Zooprofilattico Sperimentale della Sicilia, Via Gino Marinuzzi 3, 90129 Palermo, Italy; sergio.migliore@izssicilia.it (S.M.); paola.galluzzo@izssicilia.it (P.G.); galuppolucia@gmail.com (L.G.); angelica.corrente@izssicilia.it (A.C.); guidoruggero.loria@izssicilia.it (G.R.L.); roberto.puleio@izssicilia.it (R.P.); 2 Department of Biological, Chemical and Pharmaceutical Science and Technology (STEBICEF), University of Palermo, Viale delle Scienze, 90128 Palermo, Italy; 3 Department of Veterinary Medicine, University of Perugia, 06126 Perugia, Italy; elvio.lepri@unipg.it; 4 Department of Bacteriology, OIE Reference Centre for Contagious Agalactia, Animal and Plant Health Agency, Addlestone KT15 3NB, UK; anne.ridley@apha.gov.uk

**Keywords:** contagious agalactia, *Mycoplasma agalactiae*, ticks, sheep and goats, immunohistochemistry

## Abstract

*Mycoplasma agalactiae* (*Ma*) is considered the primary causative agent of contagious agalactia (CA) in sheep and goats, which causes severe losses to the small ruminant dairy industry. As early as 1816, it was thought that environmental factors played a role in pathogen maintenance in endemic areas. Specifically, recent studies hypothesized a vector role for arthropods in the epidemiology of disease. The aim of this study was to investigate the presence and anatomical localization of *Ma* in naturally infected *Riphicephalus bursa* ticks to better evaluate tick–pathogen interactions. Salivary glands and ovaries of confirmed *Ma*-positive *R. bursa* were analyzed to look for the *Ma* antigen using immunohistochemistry (IHC). IHC showed strong positivity to *Ma* in the cytoplasm of salivary cells as well as in cells from the ovary. Our work demonstrated for the first time the crossing of the tick midgut barrier by *Ma* and the subsequent infection of organs capable of spreading the infection, and this result represents an absolute novelty in disease-related knowledge. Our preliminary results provide conclusive evidence of the potential vector role represented by hard ticks in the epidemiology of CA. Further field and laboratory investigations are necessary to confirm the tick role in the transmission of clinical CA.

## 1. Introduction

*Mycoplasma agalactiae* (*Ma*) is considered the primary causative agent of contagious agalactia (CA) in small ruminants [1]. CA is a multiorgan syndrome, characterized by monolateral or bilateral mastitis and less frequently keratoconjunctivitis, arthritis, and abortion both in sheep and goats [2]. The disease spreads in farms using traditional husbandry for milk and dairy products throughout many regions of the world; it is associated with severe losses to the small ruminant dairy industries, primarily due to high morbidity in sheep and goat populations and consequent drastic fall or complete loss in milk production [3,4]. 

CA has been known of for more than 200 years, as it was first reported in 1816 in Italy, where it was recognized by experienced shepherds and veterinarians as “mal del sito” (“disease of the place”), owing to its ability to persist in an environment and contaminate newly introduced herds grazing the same pasture [4]. The potential link to ecological and environmental conditions associated with the survival of *Ma* outside the primary host and transmission in the absence of close contact with infected animals remains an important risk factor to be clarified. Ticks, mites, or other hematophagous insects may have a role in the transmission of CA, but this has not been demonstrated experimentally. Further studies are needed to confirm whether ticks and insects are capable of infecting the small ruminant host rather than just carrying the mycoplasmas (www.discontools.eu, accessed on 2 March 2024).

It is well known that ticks are efficient vectors of diseases, and a lot of pathogens can be transmitted by them, including several diseases of major zoonotic importance [5]. The biological persistence of the pathogens in the vector allows the passage of pathogens through the different stages of the tick life cycle (transstadial), vertically in the ovaries (transovarial), and sometimes within the same tick stage following interrupted feeding (intrastadial) [6]. 

Ticks are obligatory hematophagous ectoparasites, and around 900 species have been described [6]. Their survival is strongly influenced by environmental conditions as well as by their capacity to find hosts to perpetuate their life cycle [7]. In particular, hard ticks (Ixodidae) parasitize their hosts for the blood meal only; among these, the genera *Amblyomma*, *Dermacentor*, *Haemophysalis*, *Hyalomma*, and *Rhipicephalus* frequently parasitize small ruminants [8]. In the Mediterranean area, the presence of hard ticks in small ruminants can be observed throughout the year but more commonly in spring and autumn. 

Hematophagous arthropods have been proposed as reservoirs or vectors for several members of the order of Mycoplasmatales [9]; this role was first confirmed in flea and mite species [10]. Several studies have reported that hematophagous ectoparasites may represent the natural vehicle for transmission of uncultivable blood-borne hemoplasma among cats [9,11,12,13] and dogs [14,15]. In ruminants, mosquitoes and hematophagous flies have been linked to transmission of the hemoplasmas *Mycoplasma wenyonii* and “*Candidatus* Mycoplasma haemobos” [16], the latter associated with a drop in milk production, lower calf birth weight, fever, anorexia, depression, and hematuria [17,18,19,20,21]. 

In small ruminants, a minor role of flies, lice, and mosquitoes in the transmission of hemotropic *Mycoplasma ovis* [22] and *Mycoplasma mycoides* subsp*. capri* (formerly large colony-type), another causative agent of CA [10], has been proposed. Finally, a recent study reports the presence of live and viable *Ma* in *Riphicephalus bursa*, suggesting a role for this species in maintaining and spreading CA among herds and in the maintenance of endemism in the environment [23]. 

To date, transmission pathways of animal mycoplasmas as well as many other epidemiological and pathological aspects remain unclear; therefore, a multidisciplinary approach is needed to fill these gaps. The aim of this preliminary study was the use of immunohistochemistry (IHC) to detect the presence and the anatomical localization of the *Ma* antigen in naturally infected *R. bursa* ticks to investigate their role in the epidemiology of CA. For this purpose, ticks were collected from sheep in a flock in an endemic area of Sicily (Southern Italy) in which CA caused by *Ma* had been previously reported.

## 2. Materials and Methods

### 2.1. Sampling and Tick Identification

Adult ticks at various stages of engorgement were randomly collected during the spring season using fine surgical forceps from lactating sheep with a massive presence of attached parasites. Sheep were raised in a farm historically infected by *Ma* in the province of Palermo (Northwest Sicily—Italy), with approximately 200 sheep reared in traditional semi-extensive system, characterized by pastures, sometimes shared with other flocks. The last CA outbreak was resolved in 2020, and at the time of sampling, the farm was considered free of CA and vaccination protocols were in place. Contextually, to assert the negativity to *Mycoplasma* spp. infection, a sample of milk for each sampled sheep was also collected.

Sampled ticks were placed in a portable cool box and immediately transferred to the Istituto Zooprofilattico Sperimentale della Sicilia “A. Mirri” (WOAH Reference laboratory for Contagious Agalactia) for identification and *Mycoplasma* spp. detection. Before laboratory analyses, ticks were kept alive for a week at room temperature, in order to cleanse themselves of any ingested blood. Species, sex, and status identification of ticks were performed by standard morphological observations [24]. Once morphologically identified, ticks were grouped according to species, stage, and animal of origin.

### 2.2. Tick Dissection

Adult male and female ticks were placed in separate sampling tubes. Before dissection, ticks were anesthetized by thermal shock at +4 °C for one day and washed twice in distilled water and once in 70% ethanol for 5 min and then allowed to dry on a clean paper towel [25] to remove surface debris including host blood and hair or soil particles. The ticks were at first immobilized using a drop of balsam on a Petri dish in plastic and dissected lengthwise in two parts using sterile fine-tipped forceps with the tick’s dorsal surface facing up. Initial incision was made using sterile surgical blade, starting from the basis capitulum, mouth parts, and continued along soft tissue between the dorsal and ventral surfaces and slicing up the tick’s dorsum to expose the organs. PBS solution was used to move debris and visualize organs of interest. One half of the sample was screened by cultural and molecular methods and homogenized in 500 μL of *Mycoplasma* broth before sub-culturing for pathogen isolation and molecular tests. In the second half, internal organs were isolated. After removal, the salivary glands (grape-like clusters) and ovaries were gently washed with PBS to remove minimal midgut contamination. Finally, the isolated organs were placed in a special cytoinclusion box (Cytomatrix^®^, UCS Diagnostic S.r.l. & Università Campus Bio-Medico di Roma, Rome, Italy) and fixed in 10% neutral formalin. Stereomicroscope (SMZ800N, Nikon, Tokyo, Japan) was used to visualize the ticks throughout the dissection process.

### 2.3. Microbiological Analysis and Molecular Analysis 

Viable *Mycoplasma* spp. was cultured starting from 300 μL of milk sample or 300 μL of tick homogenate, transferred to a sterile bijou containing 2.7 mL of *Mycoplasma* broth medium [26] and incubated at 37 °C in 5% CO_2_ atmosphere. A reference strain of *M. agalactiae* NCTC10123 was used as a positive control and the culture broth as a negative control. After 24 h, to exclude the growth of the potential contaminating microorganisms, the broth culture was passed through a 0.45 µm filter. Following an incubation time between 48 and 72 h, 10 µL of broth was sub-cultured on *Mycoplasma* agar media and then monitored every 24 h for up to 7 days for the presence of typical Mycoplasma colonies (“fried egg”) [26].

All of the *Mycoplasma*-like colonies had their DNA extracted using the Quick-DNA Miniprep Plus Kit (Zymo Research, Irvine, CA, USA), following the manufacturer’s instructions. Culture broth, with and without *M. agalactiae* NCTC10123, was subjected to DNA extraction in order to obtain positive and negative controls, respectively, which were useful as controls in the real-time PCR. To confirm the presence of *Ma* DNA, a commercial TaqMan real-time PCR was carried out. To determine the presence of other agents responsible for CA in goats (mycoplasmas of the *M. mycoides* group), the VetMAX^TM^ *M. agalactiae* and *M. mycoides* Kit (Thermo Fisher Scientific, Waltham, MA, USA) was used. This kit contains additional checks useful for understanding the goodness of the result obtained: the EPC CAS, positive control for *M. mycoides* and for *Ma*, already extracted positive control to be amplified during real-time PCR, and the IPC CAS, an internal extraction positive control to be added to each sample in the lysis step of the nucleic acid extraction. A positive IPC result validates the extraction of samples, eliminating false negatives and verifying the effect of the potential inhibitors. Real-time PCR was performed using the CFX96 Touch Real-Time PCR Detection System (Bio-Rad, Hercules, CA, USA).

### 2.4. Histology

Ticks and dissected organ adsorbed in the cytomatrix were fixed in 10% neutral formalin. The samples were subsequently washed in tap water, dehydrated through an ascending series of alcohols (70%, 80%, 90%, 96%, and 100%), and cleared in xylene by an automatic processor (ASP300, Leica, Wetzlar, Germany) with a brief protocol for small biopsies. The half ticks and the dissected organ absorbed in the cytomatrix were embedded into paraffin blocks. The blocks were sectioned into 5 µm thick sections using microtome (RM2255, Leica, Wetzlar, Germany) and put onto standard glass slide for standard evaluation with Hematoxylin and Eosin (H&E) and in polysine adhesion for IHC staining. 

### 2.5. Hematoxylin and Eosin Staining

The sections of both ticks and isolated organs were stained with H&E by an automatic colorimeter (Shandon Varistain Gemini, Thermofisher Scientific, Waltham, MA, USA) and then examined for standard morphologic evaluation under a microscope (Leica DM LB) with a camera attachment (Nikon Optiphot-2, Nikon Coolpix P7100, Tokyo, Japan) and photographed.

### 2.6. IHC for Mycoplasma agalactiae

The sections were first dried in an oven at 56 °C for 15 min and then deparaffinized in xylol and descending series of alcohols (100%–96%–80%–70%) to distilled water. For immunohistochemical staining, slides underwent antigen retrieval with citrate buffer (pH 6) at 95 °C for 15 min, washing in distilled water, and blockage of non-specific sites by blocking buffer; IHC staining was performed using a primary monoclonal antibody 5G12 (VSD, Stormont, Belfast) (kindly donated by H. J. Ball) at a dilution of 1:2000 in PBS for 18 h at 4 °C [25]. Afterwards, the slides were incubated with a secondary antibody for 1 h at room temperature and then developed with DAB and counterstained with Carazzi’s Emallume, dehydrated in an increasing series of alcohols, clarified in xylol, and mounted with a coverslip using a Eukitt mount. For blocking buffer, a universal secondary antibody was used with the MACH 1 Universal HRP-Polymer Detection kit (Bio-optica, Milan, Italy). The negative control was performed by omitting the primary antibody and incubating with PBS.

## 3. Results

### 3.1. Ethical Statement

This study did not involve controls under EU Directive 2010 (2010/63/EU); tick removal from infested animals was below the threshold of the directive and also improved animal health. For the purpose of this study, permissions from the farmer were sought in advance for the collection of ticks from sheep.

### 3.2. Ticks Collection and Identification

A total of 13 females (6 engorged) and 7 males were collected from the perineum of five lactating sheep. All ticks collected were morphologically identified as *R. bursa* (Table 1).

### 3.3. Isolation and Molecular Identification of Mycoplasma agalactiae from Ticks

Microbiological investigations performed on milk samples confirmed the negativity of *Mycoplasma* spp. In contrast, typical *Mycoplasma*-like colonies (“fried eggs”) were cultured from two samples (10%) of each half tick from engorged female

Specific real-time PCR performed on the recovered isolates from *Mycoplasma*-like colonies confirmed *Ma* in each sample. In addition, the real-time PCR analysis using the VetMAX^TM^ *M. agalactiae* and *M. mycoides* kit also confirmed *Ma* in all isolated strains (Table 1).

### 3.4. Histological Analysis

For the morphological analysis of samples, H&E-stained slides of both half ticks and dissected salivary glands and ovaries were observed.

**Figure 1:**
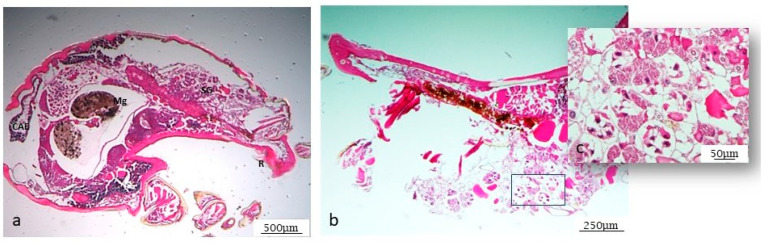
H&E-stained sagittal section of *Rhipicephalus Bursa* tick. Some structures are clearly distinguishable: (**a**) R = rostro; Mg = midgut; CAE = caecum; SG = salivary gland (2.5× magnification); (**b**) close-up of salivary gland (5× magnification); (**c**) higher magnification of the enclosed region in the box in (**b**) 10×.

**Figure 2:**
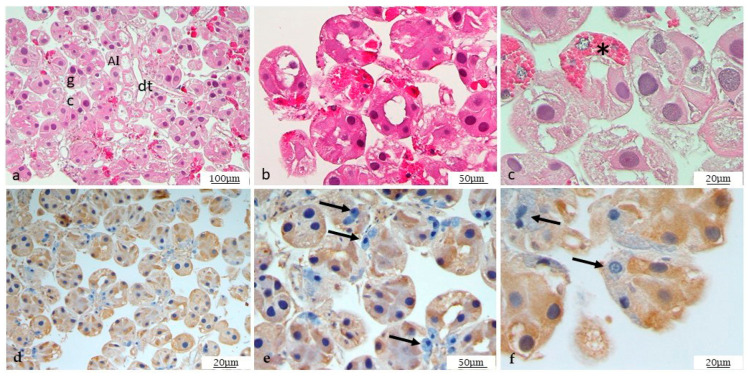
H&E-stained view of salivary glands. (**a**–**c**) In the picture, ducts and different gland cells are visible, mostly represented by type I and type II acini. dt = duct; gc = gland cells; AI = acini I; AII = acini II. (**c**) Close-up of engorged cells (*). IHC for *Ma* (**d**–**f**). Immunohistochemistry showed a high positivity, purely at the cytoplasmic level, in salivary glands, both in cells and ducts. Some cells, such as those indicated by arrows, are negative, including engorged cells.

**Figure 3:**
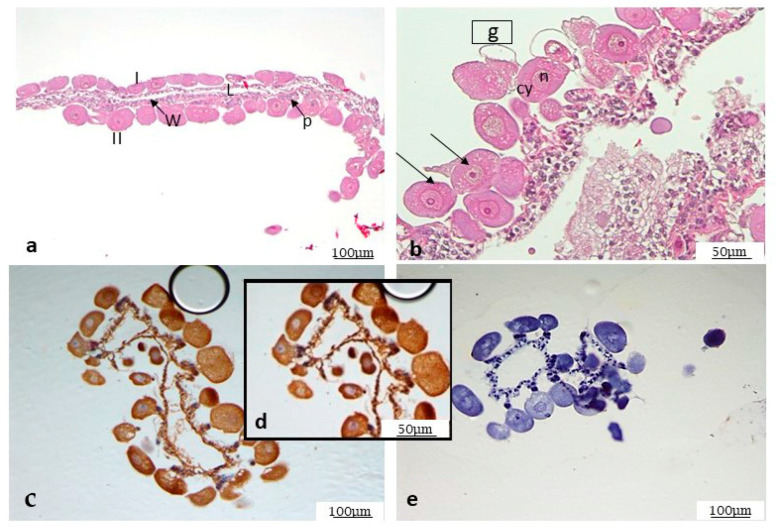
H&E-stained ovary (**a**,**b**). Two types of oocytes at stage I and II are well recognizable; L = Lumen; the arrows point to ovarian wall (W) and to pedicel (P); (**b**) close-up at a higher magnification in which it is possible to distinguish oocyte cellular compartments, arrows point indicate two oocytes in different stage. g = germinal vesicle; n = nucleolus, cy = cytoplasm (**c**–**e**). IHC for *Ma*. (**c**–**e**) A strong positivity in oocytes, again purely cytoplasmic; *R. bursa* oocyte close-up of picture in (**d**); (**e**) negative control.

Different anatomical parts of the tick are easily distinguishable, as described in Figure 1. By morphological analysis of the salivary glands, following standard staining with H&E, it was possible to observe the ducts and different gland cells, mostly represented by type I and type II acini (Figure 2a–c). In addition, some cells are still half engorged (Figure 2c), in which red blood cells are clearly distinguishable. The same procedure was also followed for the ovary. The histological analysis revealed oocytes at different development stages, as shown in Figure 3a,b, and the particular oocyte cell compartment.

### 3.5. Immunohistochemistry on Mycoplasma agalactiae Positive Ticks

Specific immunohistochemical investigations with anti-*Ma* antibody showed positivity (Table 1) at the cytoplasmic level of the salivary glands, although not all cells are positive, as shown in Figure 2e.

In ovary, most cells showed a strong positivity for *Ma* detected by IHC, which again proved to be purely cytoplasmic, as shown in Figure 3c,d. The specificity of the signal is demonstrated by the negative control, as shown in Figure 3e.

## 4. Discussion

The present study used IHC to detect and localize, for the first time, *Ma* in positive *R. bursa* ticks confirmed by isolation and PCR. Ticks were collected from sheep belonging to a farm declared free from CA at the time of this study but with confirmed historical outbreaks by *Ma* until 2020. *Ma* was detected in the salivary glands and in the ovaries of two adult females out of twenty collected ticks, demonstrating the passage through the wall of the midgut and the infection of various tick organs capable of propagating the infection to progeny and reference hosts. These results show absolute novelty in disease-related knowledge. A recent study from our team performed in three different endemic areas for CA in Sicily provided evidence of the potential involvement of ticks in the spread and maintenance of CA [23]. The data obtained in the aforementioned study showed an overall prevalence of 8.9% of viable *Ma* in *R. bursa* ticks collected from sheep and goats in three different herds, in which *Ma* infection had been previously confirmed and notified.

Sicily represents a typical Mediterranean ecosystem characterized by low or moderate rainfall and long dry seasons, in which *R. bursa* is considered the major ectoparasite of small ruminants, which spreads in hilly, marginal areas, grassy slopes or semi-desert environments [27,28]. *R. bursa* is a dual-host tick: immature stages commonly infest one host while adults infest a different one [29].

Several studies have recognized the vector role of *R. bursa* in the transmission of several pathogens, namely *Theileria* spp., *Anaplasma marginale*, *Anaplasma ovis*, *Ehrlichia canis* [30,31,32,33], *Babesia ovis* [24], and *Coxiella burnetii* [34], in ruminants. Additionally, the presence of unconventional tick-borne pathogens has been described in *R. bursa* [23,35].

The ability of the vector to establish a symbiotic relationship with a pathogen facilitating their multiplication and transmission is essential as a determinant of tick-borne disease [36]. In particular, the competence of ticks as a vector of a specific pathogen is determined by the capacity of the pathogen to survive in the tick midgut environment [37] and to reach the salivary glands where the saliva acts as the major transmission medium [38].

On the one hand, migration of the pathogen to the salivary glands would lead to survival of the pathogen after ingestion during an earlier phase of the tick’s life cycle and transmission to the next host during the next blood meal. On the other hand, the colonization of the ovary would represent the perpetuation of the infection to subsequent generations of the tick. In addition, it is well demonstrated that the pathogen transmission can occur between infected and uninfected ticks co-feeding in time or space in the absence of the host systemic infection [39]. These strategies appear similar to several tick-borne pathogens.

The detection of *Ma* in the salivary glands reported in this study suggests a high affinity of *Ma* for tick salivary glands with a consequent possibility of pathogen transmission to the host. However, further studies are needed to determine what is the minimum dose of *Ma* in the tick salivary glands to cause infection in the parasitized host. Nevertheless, it can be speculated that a sheep with massive and repeated infection by *Ma*-positive ticks may result in contracting *Ma* infection.

In this study, we also reported the presence of *Ma* in tick ovaries. The evidence of *Ma* in female reproductive organs such as the ovaries could lead to a subsequent transovarial passage which could guarantee the maintenance of the pathogen in subsequent generations.

The demonstration of *R. bursa* in the salivary glands and ovary confirmed that *Ma* is able to cross the wall of the midgut and infect various tick organs; this may indicate the potential for biological development and transmission of *Ma* in ticks. For this purpose, it would be intriguing to investigate the presence of *Ma* in other anatomical sites of infected ticks, such as hemocytes, synganglia, testes, fat bodies, and midgut.

The immunohistochemical technique employed in the present study is certainly less sensitive than that of real-time PCR in the detection of *Ma*. However, IHC has the advantage of not being compromised by cross-contamination, which is very likely during tick dissection and the isolation of organs. Furthermore, IHC is a method that allows for the identification of a specific antigen in specific cellular and subcellular compartments utilizing a specific antibody. In addition, for the inclusion of the isolated organs from dissected ticks, an innovative synthetic matrix, called Cytomatrix^®^, was used successfully in this study. The Cytomatrix^®^ matrix is characterized by a high adsorption capacity that helps to maintain the tissue architecture and three-dimensional structure of the sample being loaded onto it, reducing handling and therefore the loss of sample. This system proved to be particularly suitable for our purpose.

Since 1816, it has been believed that environmental factors play an indirect, additional role in pathogen maintenance on endemic farms for CA [4]. However, mycoplasmas are obligate parasites of cells and are poorly resistant to environmental conditions for extended periods of time [40]. Despite this, CA is endemic in Sicily and other Mediterranean countries, where unfavorable climatic conditions suggest that other factors play a role in the perpetuation of CA endemism. The distribution of CA as a climate or vector-related disease has not been generally recognized; however, keeping in mind the geographic distribution and the potential for insect or tick bites, this may be a possibility. Relatedly, climate change and the resulting colonization of new geographic areas by competent vectors could be of concern for the spread of the disease.

Our study supports this hypothesis, recognizing in the hard ticks a potential reservoir or vectors for CA agents which have not been proven to date. However, further work, including examination of other isolated anatomical parts, is required to strengthen our evidence. In addition, the reproducibility of our field results should be verified in vitro, using artificial tick feeding/infection systems. In this way, it would be possible to test the competence of several species of ticks, of different sex and stages of life (transstadial persistence), as possible vectors in the transmission and maintenance of *Ma* in areas endemic for CA. This approach could also be extended to study additional relevant diseases caused by other species of the *Mycoplasma* genus.

Our findings clearly highlight an additional risk factor in the transmission and maintenance of CA in endemic areas. In addition to the available vaccination protocols and the biosecurity measures to be applied to control the disease, it would be appropriate to think about ticks’ management in terms of both surveillance and prevention, depending on the prediction based on the presence of ticks (in which quantity and at what time of year) and climatic conditions (seasonal temperatures and precipitation). Acaricidal treatment is considered essential for the prevention of most tick-borne diseases and may be an additional control method for CA. However, its well known that the widespread use of insecticides in agriculture is a risk for promoting resistance in vectors carrying zoonotic agents and would need to be carefully assessed.

## 5. Conclusions

Our results provide further evidence of the vector competence of hard ticks, in particular *R. bursa*, in the maintenance and transmission of *Ma* in areas endemic for CA, demonstrating for the first time the crossing of the tick midgut barrier by *Ma* and the subsequent infection of other organs capable of spreading the infection.

However, the evidence of these additional environmental sources for *Ma* infection and their role in transmission and clinical disease warrant further investigation. In particular, additional field and laboratory investigations and in vitro feeding/infection studies to better evaluate tick–pathogen interactions are required.

## Figures and Tables

**Table 1:** Summary table of the results.

Sheep	*Mycoplasma* spp. Isolation from Milk	No. of Collected Ticks	Ticks Identification	*Mycoplasma* spp. Isolation from Half Ticks (No. of Positive/Total)	*Ma* Detection from Positive Samples by VETMAX^TM^ Real-Time PCR	*Ma* Antigen Detection from Positive Samples by IHC
#1	-	3	*R. bursa*	0/2	-	-
#2	-	5	*R. bursa*	2/2	2/2	2/2
#3	-	3	*R. bursa*	0/3	-	-
#4	-	4	*R. bursa*	0/1	-	-
#5	-	5	*R. bursa*	0/3	-	-
		20		2/20 (10%)		

## Data Availability

The original contributions presented in the study are included in the article, further inquiries can be directed to the corresponding author.
